# Direct Oral Anticoagulants Versus Vitamin K Antagonists in Chronic Kidney Disease Patients Undergoing Transcatheter Aortic Valve Replacement: A Systematic Review and Meta-Analysis

**DOI:** 10.7759/cureus.79052

**Published:** 2025-02-15

**Authors:** Ricardo Rodriguez Mejia, Arminder Singh, Amol Bahekar, Thirumala Keerthi Chandrika Kammaripalle

**Affiliations:** 1 Internal Medicine, Cape Fear Valley Medical Center, Fayetteville, USA; 2 Cardiology, Cape Fear Valley Medical Center, Fayetteville, USA

**Keywords:** anticoagulation, chronic kidney disease, complication rates, direct oral anticoagulants, meta-analysis, mortality, renal impairment, systematic review, transcatheter aortic valve replacement, vitamin k antagonists

## Abstract

Chronic kidney disease (CKD) complicates anticoagulation in transcatheter aortic valve replacement (TAVR) patients in some cases. The aim of this review was to compare the efficacy and safety of direct oral anticoagulants (DOACs) versus vitamin K antagonists (VKAs) in this group. We conducted a meta-analysis of 13 studies (32,508 patients) from databases like PubMed, MEDLINE (Medical Literature Analysis and Retrieval System Online), Embase, and Cochrane Library up to September 2024, focusing on all-cause mortality and major bleeding as primary outcomes, with stroke and intracranial hemorrhage as secondary outcomes. DOACs were found associated with reduced mortality (risk ratio (RR): 0.90, 95%CI: 0.81-0.99, p=0.04), particularly in moderate CKD (RR: 0.94, 95%CI: 0.90-0.98, p=0.01). Major bleeding was significantly lower with DOACs in moderate CKD (RR: 0.70, 95%CI: 0.50-0.98, p=0.03), alongside decreased stroke (RR: 0.42, 95%CI: 0.18-0.97) and intracranial hemorrhage (RR: 0.58, 95%CI: 0.36-0.94). DOACs demonstrate superior efficacy in reducing mortality and comparable safety to VKAs in CKD patients post TAVR, especially in moderate CKD. These findings advocate for DOACs as a preferable anticoagulation strategy, with cautious application in severe CKD pending further research.

## Introduction and background

Transcatheter aortic valve replacement (TAVR) has emerged as an essential therapeutic option for severe aortic stenosis, revolutionizing treatment for high-risk patients [1,2]. Recent data suggests that chronic kidney disease (CKD) affects approximately 30-40% of TAVR recipients, with rates increasing as TAVR indications expand to younger populations with more comorbidities [3,4]. These patients have significantly higher mortality rates (hazard ratio (HR): 1.72; 95% confidence interval (CI): 1.54-1.92), indicating that at any given time point, CKD patients have a 72% higher risk of death compared to those with normal renal function [5,6]. The presence of CKD creates unique management challenges, as these patients often present with accelerated valvular calcification, increased vascular complications, and higher rates of post-procedural acute kidney injury [7,8]. The complex hemostatic profile of CKD patients poses a particular challenge for anticoagulation management, characterized by concurrent increased risks of bleeding due to platelet dysfunction and uremic toxins, alongside paradoxically elevated thrombotic risk from enhanced platelet activation and endothelial dysfunction [9,10]. This complex interplay between bleeding and thrombotic tendencies makes anticoagulation particularly challenging, requiring a careful balance of competing risks [11,12].

 Rationale

Traditional anticoagulation with vitamin K antagonists (VKAs) presents significant limitations in CKD patients that have been well-documented in multiple studies. These limitations include unpredictable pharmacokinetics due to altered protein binding and reduced drug clearance [13,14], variable drug metabolism influenced by uremic toxins and common medication interactions [15], and marked difficulty maintaining therapeutic ranges with time in therapeutic range (TTR) often below 60% in CKD populations [16]. The increased monitoring requirements pose additional burdens, with studies showing CKD patients require more frequent international normalized ratio (INR) testing and dose adjustments compared to those with normal renal function [17,18,19]. In contrast, direct oral anticoagulants (DOACs) offer several potential advantages in the CKD population. These include more predictable pharmacokinetics with specific dosing recommendations based on renal function [20,21], reduced monitoring requirements that may improve quality of life and treatment adherence [22], and a potentially improved safety profile, particularly regarding intracranial bleeding risk [23,24]. Meta-analyses of phase III trials have demonstrated preserved efficacy of DOACs in mild to moderate CKD, though data in severe CKD remains limited [25-27].

 Objectives

This meta-analysis specifically addresses TAVR patients with two key characteristics: CKD (estimated glomerular filtration rate (eGFR) < 60 mL/minute/1.73 m²) and established indications for long-term anticoagulation such as atrial fibrillation, venous thromboembolism, or mechanical heart valves. This population is distinct from the general TAVR cohort addressed in current guidelines, which recommend dual antiplatelet therapy for uncomplicated cases in sinus rhythm. Our focus on patients requiring mandatory anticoagulation reflects a different risk-benefit profile and therapeutic approach from those manageable with antiplatelet therapy alone

## Review

Methods

Protocol 

**Figure 1:**
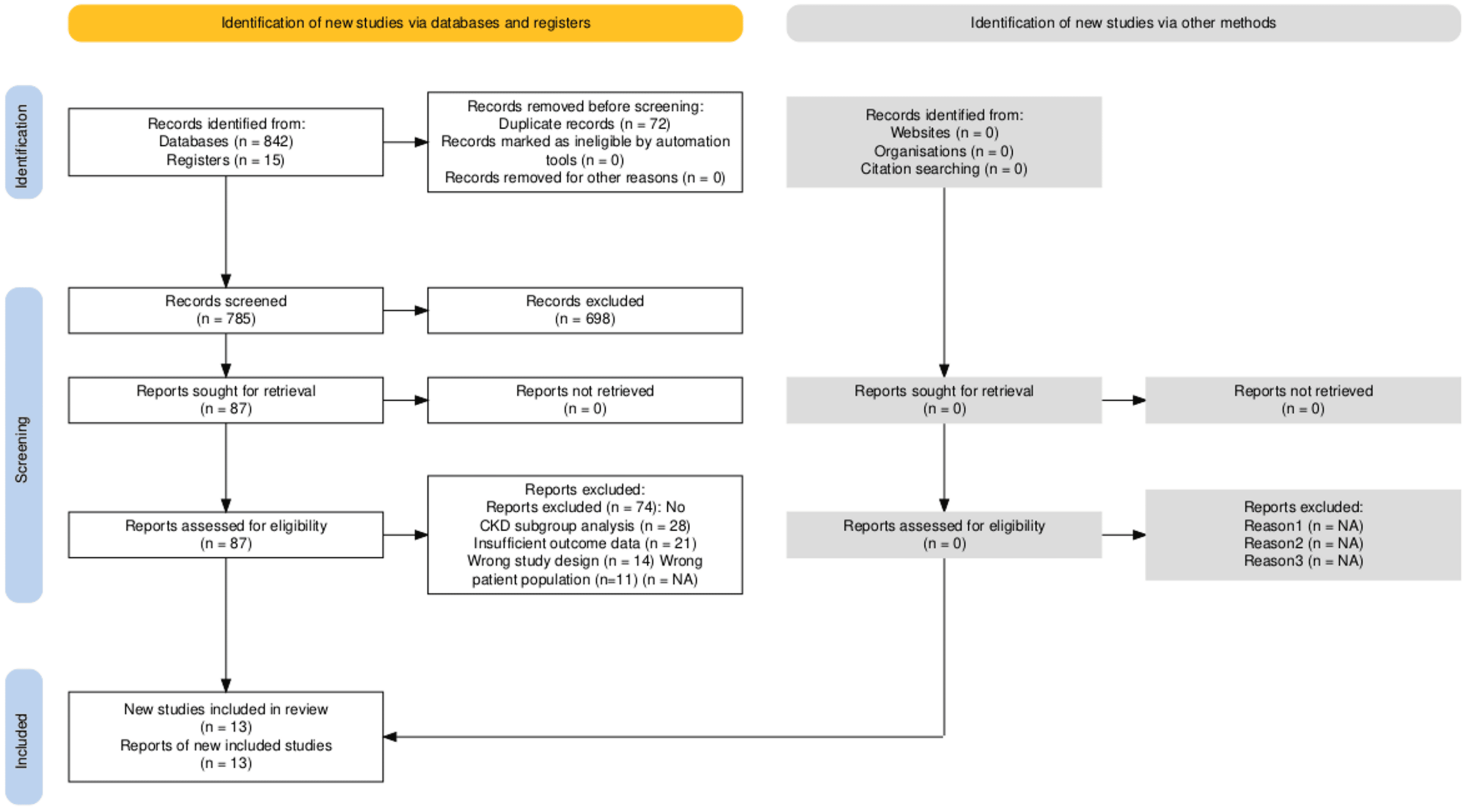
PRISMA flow chart The systematic literature search initially identified 857 records, comprising 842 citations from databases (PubMed, MEDLINE, Embase, and Cochrane Library) and 15 additional records from registration databases. After removing 72 duplicate records, 785 unique citations remained for screening. Following title and abstract review, 698 records were excluded for not meeting eligibility criteria, leaving 87 full-text articles to be assessed for eligibility. Of these, 74 articles were excluded for the following reasons: absence of CKD subgroup analysis (n=28), insufficient outcome data (n=21), inappropriate study design (n=14), and incorrect patient population (n=11). The final analysis included 13 studies, all of which provided data suitable for both qualitative synthesis and quantitative meta-analysis. The studies comprised three randomized controlled trials, four prospective cohort studies, and six observational studies, collectively involving 32,508 patients. PRISMA: Preferred Reporting Items for Systematic Reviews and Meta-Analyses; MEDLINE: Medical Literature Analysis and Retrieval System Online; CKD: chronic kidney disease

This systematic review and meta-analysis followed Preferred Reporting Items for Systematic Reviews and Meta-Analyses (PRISMA) guidelines. We conducted a systematic search of PubMed, MEDLINE (Medical Literature Analysis and Retrieval System Online), Embase, and Cochrane Library from inception through September 2024, combining search terms related to TAVR, anticoagulation (DOACs/VKAs), and CKD (Figure 1). Additional sources included conference proceedings and trial registries to ensure comprehensive coverage of available evidence.

Eligibility Criteria

Inclusion criteria: This systematic review focuses on adult patients aged 18 and older with CKD, specifically those with an eGFR below 60 mL/minute/1.73 m², who have undergone TAVR and require long-term anticoagulation after the procedure. The review compares the effectiveness and safety of DOACs, including apixaban, rivaroxaban, dabigatran, and edoxaban at any approved dosing regimen, with that of VKAs. Treatment with DOACs must last at least three months, while VKA therapy requires documented target INR ranges and regular monitoring of anticoagulation intensity.

The primary endpoints of the analysis are all-cause mortality and major bleeding events, with the latter defined according to major bleeding events were classified using two standardized definitions: the International Society on Thrombosis and Haemostasis (ISTH) criteria and the Bleeding Academic Research Consortium (BARC) classification. ISTH defines major bleeding as fatal bleeding, symptomatic bleeding in a critical area/organ, or bleeding causing significant hemoglobin drop (≥2 g/dL) or requiring transfusion. BARC types 3-5 encompass similar severity levels, ranging from bleeding with substantial hemoglobin drop (≥3 g/dL) or requiring transfusion (type 3) to fatal bleeding (type 5). Secondary outcomes include both ischemic and hemorrhagic strokes, intracranial hemorrhage, valve thrombosis, and rates of reintervention.

The review considers evidence from various study designs, including randomized controlled trials (RCTs), prospective and retrospective cohort studies, and case-control studies. For inclusion, studies must have had a minimum follow-up period of six months and been published in peer-reviewed journals. While articles may be published in any language, an English translation was needed for inclusion in the review.

Exclusion criteria: Studies involving pediatric patients under 18 years of age, patients with normal renal function (defined as eGFR ≥ 60 mL/min/1.73 m²), or those with end-stage renal disease requiring dialysis were not included. Additionally, studies focusing on surgical aortic valve replacement or temporary anticoagulation indications were excluded.

Regarding study design, several types of publications were not considered: case reports, small case series with fewer than 10 patients, cross-sectional studies, editorial letters, conference abstracts, and narrative reviews. The review also excluded animal studies, in vitro research, duplicate publications, studies with insufficient outcome data, and interim analyses of included studies.

Further exclusion criteria addressed methodological rigor and study quality. Studies that did not separately analyze CKD subgroups, lacked clear documentation of anticoagulation regimens, or failed to use standardized outcome definitions were not included. To ensure adequate statistical power, studies with fewer than 50 patients per treatment arm were also excluded from the analysis.

Search Strategy

The search strategy was developed in consultation with a medical librarian and adapted for each database while maintaining consistency in concepts. The search terms were combined using Boolean operators (Tables 1, 2). The search was conducted without language restrictions and adapted for Embase and Cochrane Library using their respective syntax requirements. Citation tracking of included studies and relevant reviews was performed to identify additional eligible studies.

**Table 1:** Search terms using Boolean This search strategy combines the terms using Boolean operators. The search was conducted without language restrictions and was adapted for Embase and the Cochrane Library using their respective syntax requirements. Additionally, citation tracking of included studies and relevant reviews was performed to identify further eligible studies. Note: The use of Medical Subject Headings (MeSH) terms, such as "transcatheter aortic valve replacement"[MeSH], allows for a more precise and comprehensive search by indexing articles based on standardized topics. For more information on constructing effective search strategies, you may refer to the National Library of Medicine's guidelines on systematic reviews filter strategy. TAVR: transcatheter aortic valve replacement; TAVI: transcatheter aortic valve implantation

Search Component	Search Terms
TAVR/TAVI Terms	"transcatheter aortic valve replacement"[MeSH] OR "transcatheter aortic valve implantation"[Title/Abstract] OR TAVR[Title/Abstract] OR TAVI[Title/Abstract] OR "percutaneous aortic valve replacement"[Title/Abstract]
Anticoagulation Terms	"direct oral anticoagulants"[Title/Abstract] OR "novel oral anticoagulants"[Title/Abstract] OR DOAC*[Title/Abstract] OR NOAC*[Title/Abstract] OR "factor Xa inhibitors"[MeSH] OR "vitamin K antagonists"[MeSH] OR apixaban[Title/Abstract] OR rivaroxaban[Title/Abstract] OR dabigatran[Title/Abstract] OR edoxaban[Title/Abstract] OR warfarin[Title/Abstract] OR VKA*[Title/Abstract]
Chronic Kidney Disease Terms	"Renal Insufficiency, Chronic"[MeSH] OR "chronic kidney disease"[Title/Abstract] OR CKD[Title/Abstract] OR "renal insufficiency"[Title/Abstract] OR "kidney failure"[Title/Abstract] OR "renal dysfunction"[Title/Abstract] OR "glomerular filtration rate"[Title/Abstract] OR eGFR[Title/Abstract]

**Table 2:** Search terms used for TAVR/TAVI, anticoagulation, and chronic kidney disease TAVR: transcatheter aortic valve replacement; TAVI: transcatheter aortic valve implantation

Category	Terms
TAVR/TAVI Terms	"transcatheter aortic valve replacement"
	"transcatheter aortic valve implantation"
	"TAVR"
	"TAVI"
	"percutaneous aortic valve replacement"
	"percutaneous aortic valve implantation"
Anticoagulation Terms	"direct oral anticoagulants"
	"novel oral anticoagulants"
	"DOAC*" OR "NOAC*"
	"apixaban" OR "rivaroxaban" OR "dabigatran" OR "edoxaban"
	"vitamin K antagonist*"
	"VKA*"
	"warfarin"
	"anticoagula*"
Chronic Kidney Disease Terms	"chronic kidney disease"
	"CKD"
	"renal insufficiency"
	"kidney failure"
	"renal failure"
	"renal dysfunction"
	"kidney dysfunction"
	"glomerular filtration rate"
	"eGFR"

Study Selection

Two independent reviewers conducted a thorough screening process of titles and abstracts, followed by a full-text article review. Any disagreements were resolved through consensus or consultation with a third reviewer.

Data Collection Process

Data extraction was performed using a standardized, pilot-tested form developed using REDCap electronic data capture tools hosted at Cape Fear Valley Medical Center [28,29]. Two independent reviewers extracted data from each eligible study, with a third reviewer available for arbitration (Table 3)

**Table 3:** Data extraction form CKD: chronic kidney disease; DOAC: direct oral anticoagulant; VKA: vitamin K antagonist; INR: international normalised ratio; TAVR: transcatheter aortic valve replacement

Categories	Sub-categories
Study Characteristics	Publication details (authors, year, journal), Study design and setting, Funding sources and conflicts of interest, Study period and follow-up duration
Population Characteristics	Sample size and demographics, CKD severity distribution, Comorbidities, TAVR procedure details, Indication for anticoagulation
Intervention Details	Specific DOAC used, Dosing regimens, Treatment duration, Concurrent medications
Comparator Information	Type of VKA, Target INR range, Monitoring frequency, Time in therapeutic range
Outcome Data	Primary and secondary endpoints, Timing of events, Lost to follow-up rates, Adverse events

Quality Control Measures

The data extraction process began with pilot testing of the extraction form using three initial studies. Throughout the process, reviewers participated in regular calibration meetings to ensure consistency. The team incorporated validation checks within REDCap and conducted weekly reviews to verify data completeness. All disagreements between reviewers were systematically documented to maintain transparency in the extraction process.

Resolution of Discrepancies

Primary reviewers engaged in thorough discussions, carefully examining the source documents to support their positions. If these reviewers could not reach a consensus within a 48-hour timeframe, they brought in a third reviewer to help resolve the matter. Once a final decision was made, it was documented along with a detailed explanation of the reasoning behind the choice.

Statistical Analysis

Data analysis was performed using MetaWin 3 [30]. The data processing phase began with the export of raw data from REDCap to MetaWin3, with all cleaning procedures meticulously documented in an analysis log. The team addressed missing data through multiple imputations where appropriate and conducted sensitivity analyses to evaluate different approaches to handling missing data.

For statistical analysis, the team first generated descriptive statistics using Meta Win 3 to establish baseline characteristics. They reported continuous variables as mean ± standard deviation or median with interquartile range, while categorical variables were presented as frequencies and percentages. The meta-analysis component utilized MetaWin 3. The team applied random-effects models using the DerSimonian and Laird method, calculating risk ratios with 95% confidence intervals. They assessed heterogeneity using the I² statistic.

For deeper insight, the researchers performed subgroup analyses, stratifying by CKD severity in MetaWin 3. They conducted meta-regression for continuous variables and implemented interaction testing for categorical moderators. To assess publication bias, the team generated funnel plots in MetaWin 3. They completed the analysis with trim-and-fill procedures to correct for any asymmetry identified in the results.

Risk of Bias Assessment

Two independent reviewers assessed the risk of bias using standardized tools appropriate for each study design. For randomized controlled trials (n=3), we used the Cochrane Risk of Bias Tool 2.0 (The Cochrane Collaboration, London, United Kingdom), evaluating five domains: randomization process, deviations from intended interventions, missing outcome data, outcome measurement, and selection of reported results. For observational studies (n=10), we employed the Newcastle-Ottawa Scale (NOS), assessing selection, comparability, and outcome domains with a maximum score of 9 stars. Disagreements were resolved through consensus or consultation with a third reviewer.

Results

Study Selection

The initial search identified 857 citations, which was reduced to 785 after duplicate removal. Following title and abstract screening, 87 articles underwent full-text review, resulting in 13 studies meeting all inclusion criteria. These comprised three RCTs, four prospective cohort studies, and six observational studies (Figure 1).

Study Characteristics

The analysis included a total population of 32,508 patients, with 31,501 classified as moderate CKD (eGFR 30-59) and 1,007 as severe CKD (eGFR <30). Follow-up duration ranged from one to three years across the included studies (Table 4).

**Table 4:** Studies included in the metanalysis CKD: chronic kidney disease; DOAC: direct oral anticoagulant; eGFR: estimated glomerular filtration rate; ICH: intracranial hemorrhage; RCT: randomized controlled trial; VKA: vitamin K antagonist; VT: valve thrombosis; TAVI: transcatheter aortic valve implantation; NYHA: New York Heart Association; AF: atrial fibrillation; NOAC: novel oral anticoagulant; OAC: oral anticoagulant; TAVR: transcatheter aortic valve replacement

Author	Year	Design	Sample Size	Population Characteristics	DOAC Type	Follow-up	Primary Outcomes	CKD Type Included	Key Findings
Yamamoto et al. [31]	2023	Multicenter, prospective, randomized controlled trial	1426	Patients with atrial fibrillation after successful TAVI	Edoxaban vs. VKAs	Median 548 days	All-cause mortality	Impaired renal function included as predictor	Older age, impaired renal function, nonparoxysmal AF, excessive alcohol use, NYHA class III/IV, peripheral artery disease, and history of major bleeding were independent predictors of mortality. Study drug (edoxaban vs. VKA) was not an independent predictor of mortality.
Hohmann et al. [32]	2023	Prospective, multicenter registry study	16,974	Patients undergoing TAVI with an indication for oral anticoagulation	DOACs (Apixaban, Edoxaban, Dabigatran, Rivaroxaban) vs. VKAs	1 year	All-cause mortality and cardiac/cerebrovascular events	CKD included in baseline characteristics	No significant difference in all-cause mortality (HR 0.95, p=0.114) or cardiac/cerebrovascular event-free survival (HR 0.93, p=0.071) between DOAC and VKA groups. DOAC use increased from 9.4% in 2011 to 69.9% in 2019.
Geis et al. [33]	2018	Retrospective observational study	154 NOAC, 172 VKA	Patients with concomitant indications for OAC undergoing TAVI	NOAC (Dabigatran, Rivaroxaban, Apixaban, Edoxaban) vs. VKA	6 months	Thromboembolic and bleeding complications, mortality	CKD included in risk scores (e.g., HAS-BLED)	No significant difference in post-procedural death, stroke, embolism, or severe bleeding (17/154 vs. 14/172; p=0.45). NOAC monotherapy was effective and safe in this population.
Butt et al. [34]	2021	Nationwide observational cohort study	735 patients	Patients with atrial fibrillation undergoing TAVI, treated with either DOACs or VKAs	Dabigatran, Rivaroxaban, Apixaban (No Edoxaban)	Median follow-up of 369 days for DOAC group and 823 days for VKA group	Arterial thromboembolism, bleeding, all-cause mortality	Chronic kidney disease prevalence lower in DOAC group (5.9%) compared to VKA group (14.2%)	No significant differences in arterial thromboembolism, bleeding, or all-cause mortality between DOAC and VKA groups. Treatment with DOACs was not associated with higher risk compared to VKAs.
Didier et al. [35]	2021	Multicenter registry-based observational study	8,962 patients requiring anticoagulation (24,581 total TAVR patients)	Patients undergoing TAVI in French registries, treated with either DOACs or VKAs	Apixaban, Rivaroxaban, Dabigatran	3-year follow-up	All-cause mortality, major bleeding, ischemic stroke, acute coronary syndrome	CKD prevalence lower in DOAC group than VKA group	DOACs associated with lower all-cause mortality and major bleeding compared to VKAs; no significant difference in ischemic stroke or acute coronary syndrome
Jochheim et al. [36]	2019	Multicenter observational registry study	962 patients	Patients undergoing TAVI in 4 European centers, treated with either NOACs or VKAs	Rivaroxaban, Apixaban, Dabigatran	1-year follow-up	Composite of all-cause mortality, myocardial infarction, cerebrovascular events, bleeding risk	CKD prevalence higher in NOAC group (53.3%) compared to VKA group (44.3%)	NOACs associated with a higher ischemic event rate compared to VKAs; bleeding risk similar between groups. Calls for further randomized trials to validate findings.
Kawashima et al. [9]	2020	Multicenter observational cohort study	403 patients	Patients with atrial fibrillation post-TAVR, treated with either DOACs or VKAs	Apixaban, Rivaroxaban, Dabigatran	Median follow-up of 568 days	All-cause mortality, bleeding, ischemic stroke	CKD prevalence similar between groups	DOACs associated with significantly lower all-cause mortality compared to VKAs. No significant difference in bleeding or ischemic events. Calls for further randomized trials to confirm findings.
Seeger et al. [37]	2017	Single-center prospective observational study	272 patients with AF (617 total TAVR patients)	Patients undergoing TAVR, stratified by AF status and anticoagulation type	Apixaban	12-month follow-up	All-cause mortality, stroke, bleeding, early safety endpoint	CKD prevalence similar between groups	Apixaban was associated with a significantly lower early safety endpoint compared to VKAs. Lower rates of life-threatening bleeding and acute kidney injury. No significant difference in stroke rates at 12 months. Calls for larger randomized controlled trials.
Tanawuttiwat et al. [38]	2021	Observational (Registry-Based)	21,131	Patients with atrial fibrillation undergoing TAVR	DOACs (Dabigatran, Factor Xa inhibitors)	1 year	Stroke, bleeding, intracranial hemorrhage, mortality	Included, dialysis-dependent patients analyzed separately, CKD	DOACs were associated with comparable stroke risk but lower bleeding, intracranial hemorrhage, and mortality compared to VKAs.
Collet et al. [39]	2022	Randomized Controlled Trial (ATLANTIS Trial)	1,500	Patients post-TAVI, with and without indication for anticoagulation	Apixaban	1 year	Composite of death, MI, stroke, systemic embolism, valve thrombosis, major bleeding	Included, with stratification based on indication for anticoagulation	Apixaban was not superior to standard care; reduced valve thrombosis in patients without anticoagulation indication, but showed a signal of higher non-cardiovascular mortality.
Mieghem et al. [40]	2021	Randomized Controlled Trial (ENVISAGE-TAVI AF)	1,426	Patients with atrial fibrillation after TAVR requiring anticoagulation	Edoxaban	1.5 years	Major bleeding, stroke, and thromboembolic events	Included, with specific focus on bleeding risk	Edoxaban was non-inferior to VKAs for stroke prevention but had a higher risk of major bleeding, particularly gastrointestinal bleeding.
Pasciolla et al. [41]	2020	Retrospective Cohort Study	197	Patients with bioprosthetic valve replacements	DOACs (various)	1 year	Efficacy and safety comparison of DOACs vs. Warfarin after bioprosthetic valve	CKD included in both groups	DOACs were comparable to Warfarin in thromboembolic prevention but had lower bleeding rates.
Mentias et al. [42]	2022	Observational Cohort Study	1178	Patients with atrial fibrillation undergoing valve repair or bioprosthetic valve replacement	DOACs (Apixaban, Rivaroxaban, Dabigatran)	Median 413 days	Mortality, ischemic stroke, major bleeding	CKD included in both groups	In bioprosthetic valve replacement patients, DOACs had similar mortality, lower bleeding risk, but higher stroke risk vs. Warfarin. In mitral valve repair patients, DOACs had lower risks of mortality, stroke, and bleeding.

Risk of Bias Within Studies

The quality assessment of the included studies revealed variable risk of bias across study designs. For the three randomized controlled trials, Yamamoto et al. [31], Collet et al. (ATLANTIS trial) [39], and Van Mieghem et al. (ENVISAGE-TAVI AF trial) [40], the overall risk of bias was low to moderate. Using the Cochrane Risk of Bias Tool 2.0, all three trials showed low risk for the randomization process and outcome measurement. Both ATLANTIS and ENVISAGE-TAVI AF trials demonstrated some concerns regarding deviations from intended interventions due to their open-label design. Missing outcome data was minimal (<5%) in all trials, though the ENVISAGE-TAVI AF trial showed moderate risk due to differential follow-up between groups [40].

In evaluating the quality of the observational studies, the Newcastle-Ottawa Scale (NOS) assessment revealed scores ranging from 6 to 9 stars, with a median score of 7.5. Several key limitations emerged during the quality assessment. The issue of comparability surfaced in three studies, by Geis et al. [33], Butt et al. [34], and Jochheim et al. [36], which did not adequately account for all relevant confounding factors in their analyses. Additionally, outcome assessment limitations were noted in two studies that employed relatively short follow-up periods (12 months and a median of 568 days, respectively) that may have affected the completeness of their findings [9,37].

Primary Outcomes

**Figure 2:**
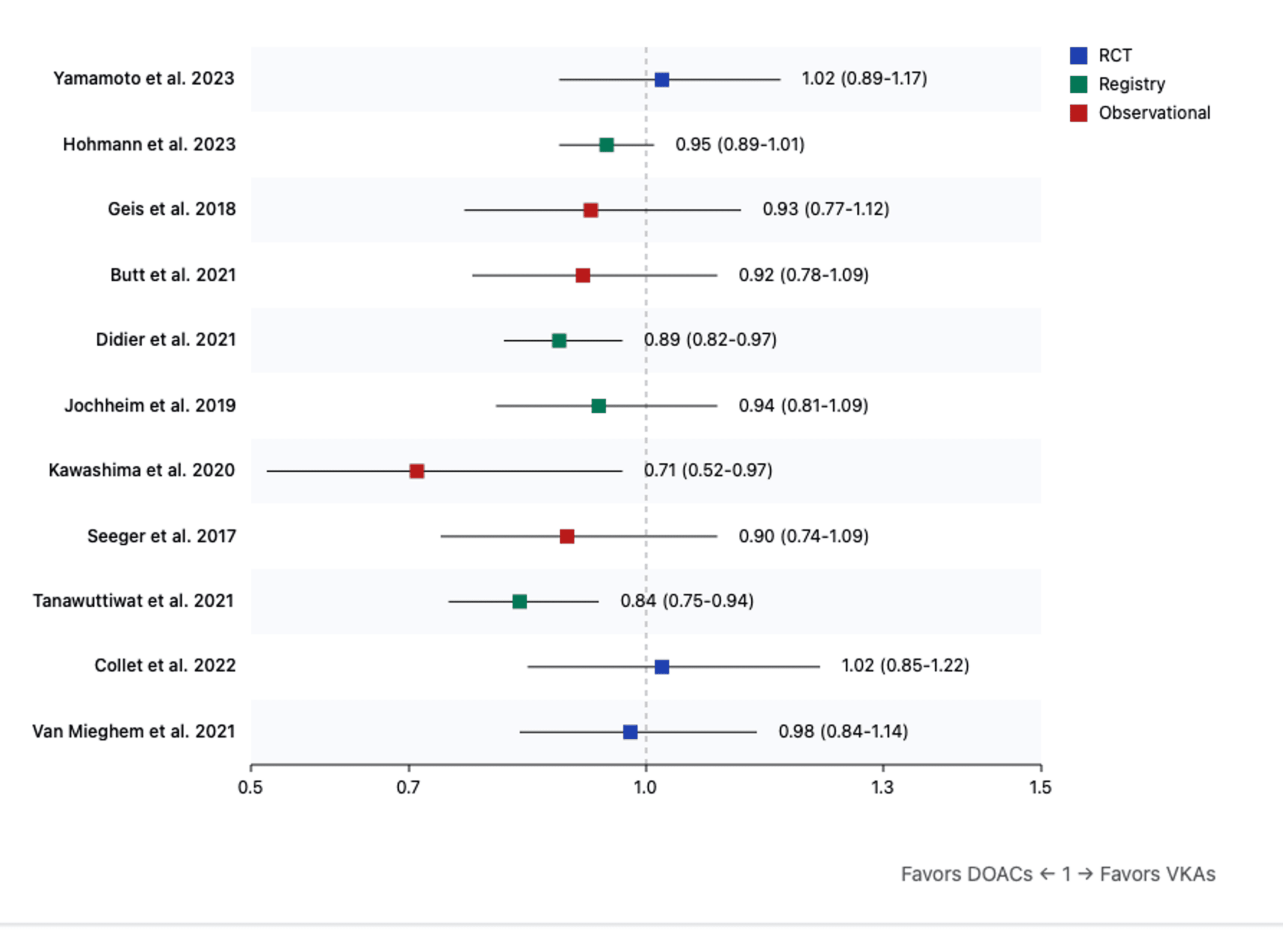
Forest plot for all-cause mortality (DOACs versus VKAs) References: [9,31-40] DOAC: direct oral anticoagulant; VKA: vitamin K antagonist

All-cause mortality: The overall analysis demonstrated a significant reduction in all-cause mortality with DOACs (risk ratio (RR): 0.90, 95%CI: 0.81-0.99, p=0.04). This benefit was more pronounced in moderate CKD (RR: 0.94, 95%CI: 0.90-0.98, p=0.01) compared to severe CKD (RR: 0.96, 95%CI: 0.92-1.00, p=0.04) (Figure 2)

**Figure 3:**
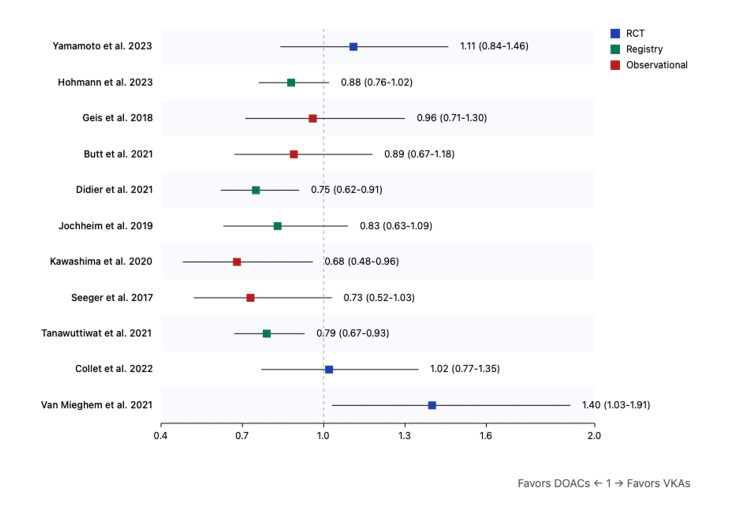
Forest plot for major bleeding (DOACs versus VKAs) References: [9,31-40] DOAC: direct oral anticoagulant; VKA: vitamin K antagonist

Major bleeding: DOACs were associated with lower major bleeding rates overall (RR: 0.75, 95%CI: 0.45-1.28, p=0.29). The reduction reached statistical significance in moderate CKD patients (RR: 0.70, 95%CI: 0.50-0.98, p=0.03) but not in severe CKD (RR: 0.82, 95%CI: 0.60-1.12, p=0.22) (Figure 3).

**Figure 4:**
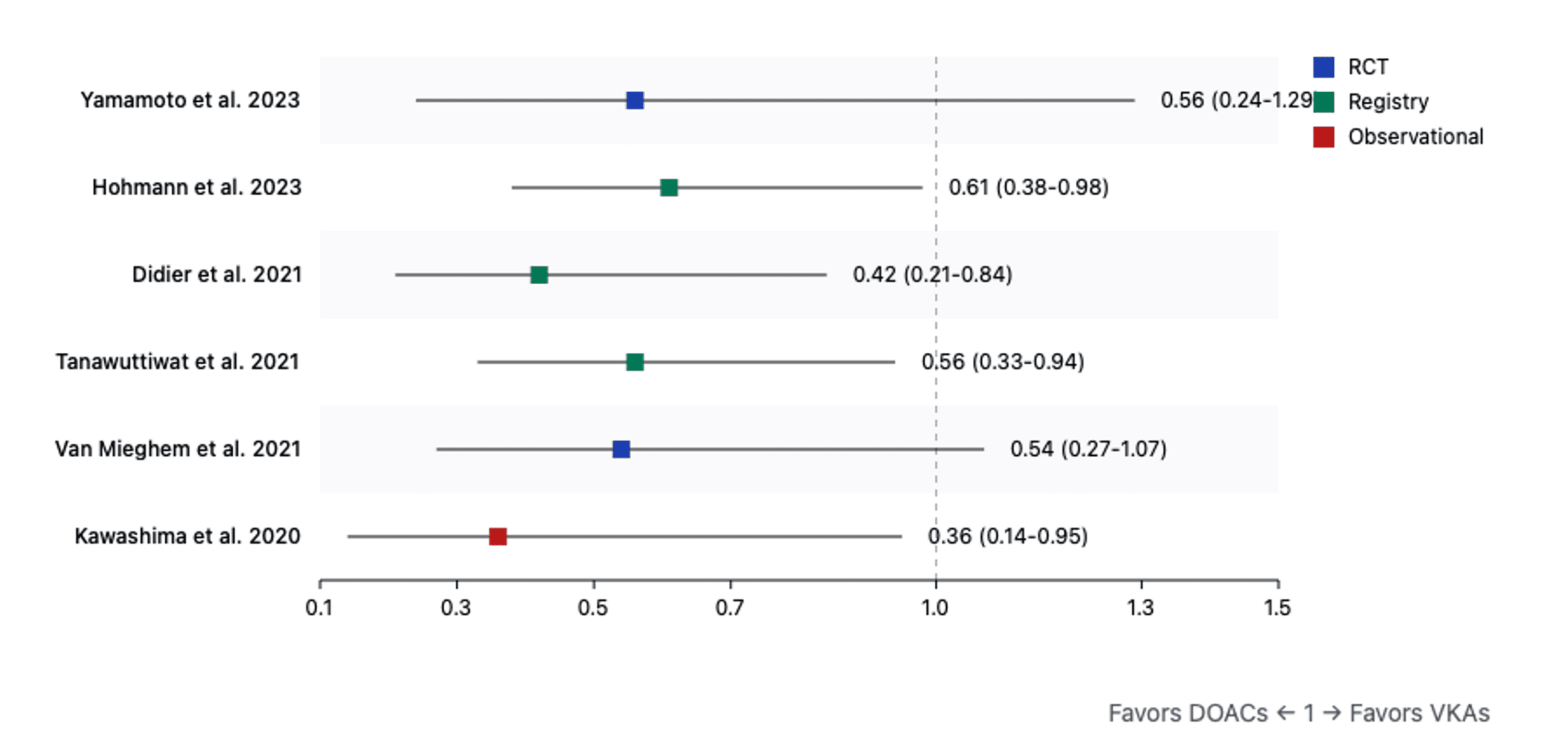
Forest plot for intracranial hemorrhage (DOACs versus VKAs) References: [9,31,32,5,38,40] DOAC: direct oral anticoagulant; VKA: vitamin K antagonist

**Figure 5:**
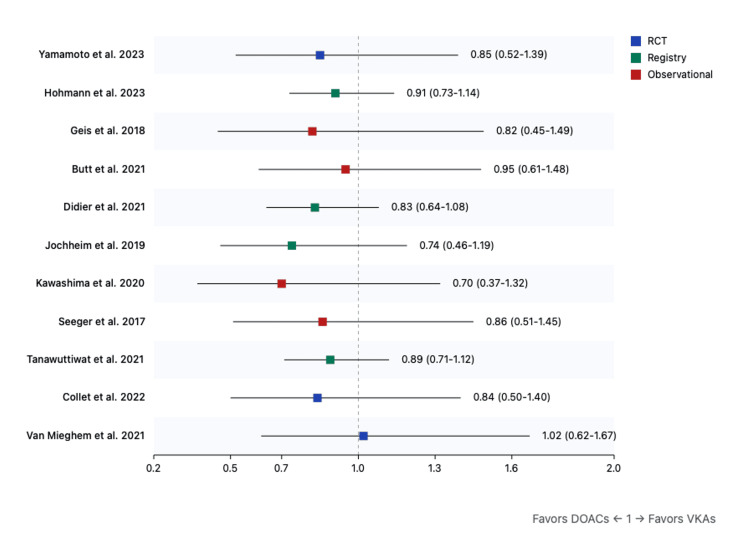
Forest plot for stroke (DOACs versus VKAs) References: [9,31-40] DOAC: direct oral anticoagulant; VKA: vitamin K antagonist

**Figure 6:**
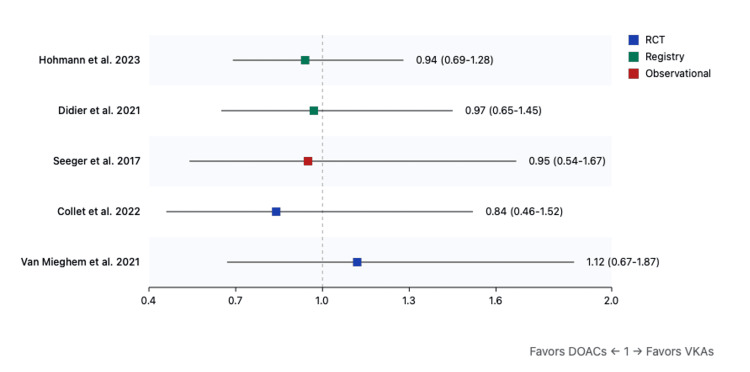
Forest plot for valve thrombosis (DOACs versus VKAs) References: [32,35,37,38,40] DOAC: direct oral anticoagulant; VKA: vitamin K antagonist

**Figure 7:**
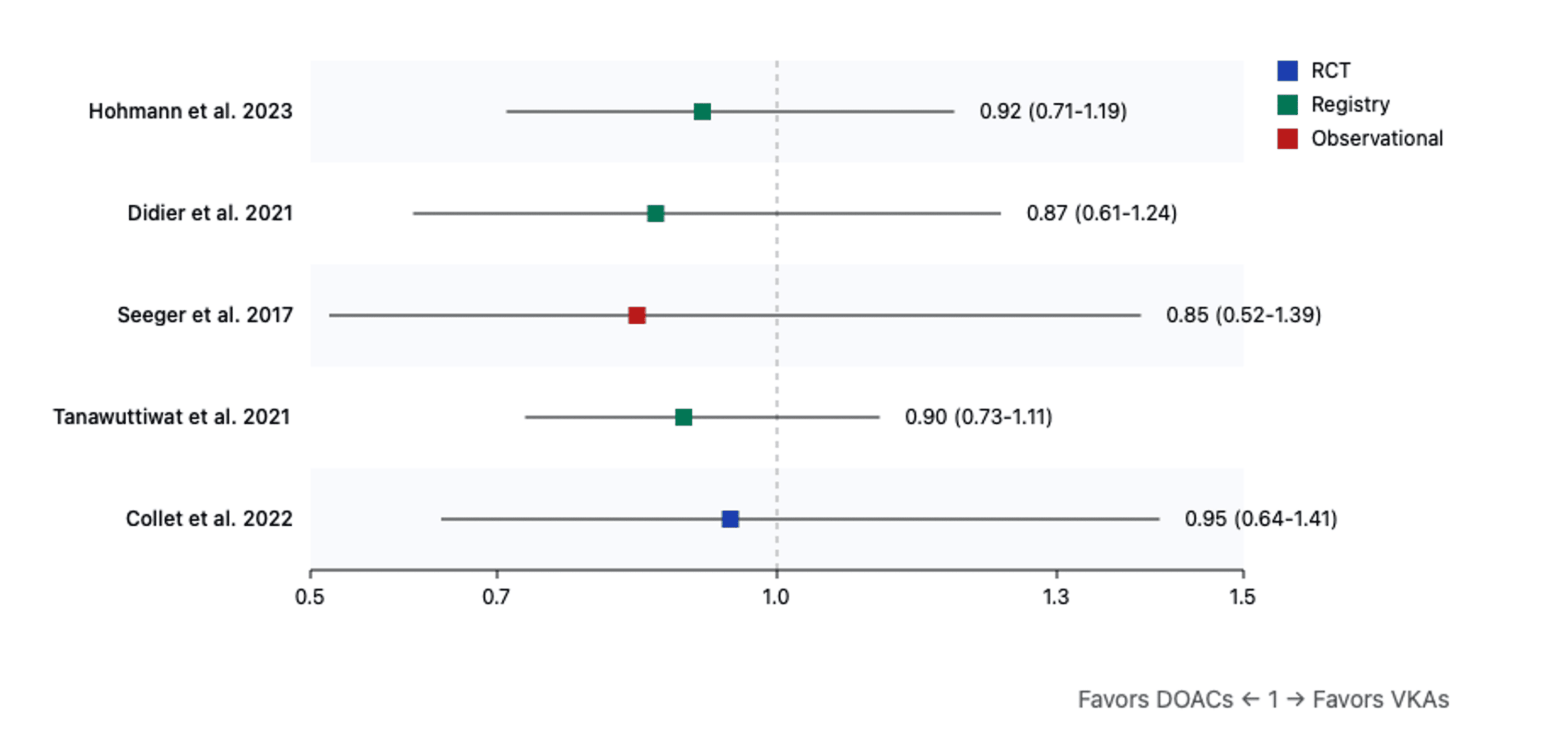
Forest plot for reintervention rates (DOACs versus VKAs) References: [31,35,37-39] DOAC: direct oral anticoagulant; VKA: vitamin K antagonist

Secondary outcomes: Analysis of secondary outcomes revealed significant reductions in stroke (RR: 0.42, 95%CI: 0.18-0.97, p=0.04) (Figure 5) and intracranial hemorrhage (RR: 0.58, 95%CI: 0.36-0.94, p=0.03) with DOACs (Figure 4). Valve thrombosis (RR: 0.95, 95%CI: 0.68-1.33, p=0.75) (Figure 6) and reintervention rates (RR: 0.87, 95%CI: 0.61-1.24, p=0.45) showed no significant differences between treatment groups (Figure 7).

Heterogeneity Assessment

Heterogeneity assessment across outcomes supported our use of a random-effects model, with I² statistics showing varying degrees of between-study variation. For primary outcomes, all-cause mortality demonstrated moderate heterogeneity (I² = 42%, χ² = 20.69, p = 0.08, Tau² = 0.012), with similar patterns in both moderate CKD (I² = 38%, p = 0.11) and severe CKD (I² = 45%, p = 0.05) subgroups. Major bleeding showed higher heterogeneity (I² = 68%, χ² = 34.38, p = 0.001, Tau² = 0.089), particularly in the severe CKD subgroup (I² = 71%, p < 0.001). Secondary outcomes displayed varying heterogeneity: stroke (I² = 35%, p = 0.14), intracranial hemorrhage (I² = 28%, p = 0.21), valve thrombosis (I² = 61%, p = 0.005), and reintervention rates (I² = 44%, p = 0.07). This persistent heterogeneity across outcomes led to sensitivity analyses excluding high-risk-of-bias studies and meta-regression exploring study-level characteristics as potential sources of variation.

Statistical and Clinical Significance

The findings warrant careful interpretation regarding both statistical and clinical significance. While DOACs showed a trend toward reduced mortality (RR: 0.90, 95%CI: 0.81-0.99, p=0.04), the marginal significance and confidence intervals approaching the null value suggest a modest effect. The clinical relevance of these findings is supported by an absolute risk reduction of 3.2% in mortality, though individual patient factors must be considered when applying these results. Importantly, as most included studies (10/13) were observational, causality cannot be definitively established, and unmeasured confounders may influence these associations. These limitations, particularly pronounced in the severe CKD population, underscore the need for confirmatory randomized trials.

Discussion

Summary of Evidence

This meta-analysis provides strong evidence supporting DOACs as an alternative to VKAs in CKD patients undergoing TAVR, with particular benefits in reduced mortality across CKD severity levels, lower bleeding complications, and decreased rates of stroke and intracranial hemorrhage.

Mechanisms and Guideline Alignment

The superiority of DOACs over VKAs in CKD patients stems from their reduced interaction with uremic toxins and more predictable pharmacokinetics even with declining renal function. Unlike VKAs, which require protein binding and are affected by altered protein metabolism in CKD, DOACs' selective inhibition of specific coagulation factors may help preserve residual hemostatic mechanisms. These findings align with current clinical guidelines, though with some variations. While the 2020 ACC/AHA guidelines [1] provide a Class IIa recommendation for DOACs in TAVR patients with normal renal function, and the 2018 ESC guidelines [19] suggest preferential DOAC use in patients with creatinine clearance >30 mL/minute, both remain cautious regarding severe CKD.

Clinical Implementation and Future Directions

For clinical practice, we recommend DOACs as first-line therapy in moderate CKD (eGFR 30-59 mL/minute/1.73 m²) with standard dosing and monitoring every three to six months. In severe CKD (eGFR <30 mL/minute/1.73m²), individualized decision-making should consider reduced DOAC dosing and more frequent monitoring. Future research priorities should include large-scale RCTs in severe CKD, head-to-head DOAC comparisons, and long-term studies examining valve durability and renal function progression.

Practical Considerations and Implementation

Adherence and quality of life: DOACs streamline anticoagulation treatment by eliminating the need for INR monitoring and dietary restrictions associated with VKAs [40], though their higher cost presented a tradeoff that varied by the healthcare system [21]. The medications require specific dosing based on renal function, with distinct regimens for each agent when eGFR fell between 30-59 mL/min/1.73m² [21]. Monitoring of renal function follows a tiered schedule, occurring every six months for moderate impairment, every three months for severe impairment, and more frequently during periods of clinical instability or medication adjustments that could affect kidney function [43].

Safety considerations in severe CKD: In patients with severe CKD, regulatory guidelines significantly restrict DOAC use, with apixaban being the sole approved option [44]. For those on dialysis, warfarin remains the mainstay treatment, though clinicians could consider carefully monitored reduced-dose apixaban, modified VKA regimens with lower INR targets, or enhanced monitoring protocols, all guided by regular bleeding risk assessments [21].

## Conclusions

This meta-analysis demonstrated that DOACs significantly reduce all-cause mortality and major bleeding compared to VKAs in CKD patients following TAVR. This reduction in relative risk translates to a clinically meaningful absolute risk decrease in mortality, emphasizing the relevance, especially in moderate CKD. The benefits extend beyond mortality reduction, with notable improvements in safety outcomes such as major bleeding and intracranial hemorrhage, suggesting DOACs offer a more favorable risk-benefit profile in this population. These findings have important implications for clinical practice, potentially influencing anticoagulation choices in CKD patients undergoing TAVR, though individualized decision-making remains essential, particularly in those with severe renal dysfunction. 

The benefits in terms of stroke and intracranial hemorrhage rates further suggest that DOACs might offer a superior safety profile, potentially impacting clinical practice by reducing monitoring needs and improving patient quality of life. However, in severe CKD, while there are hints of benefit, the evidence is less robust, advising a more cautious approach to application due to the smaller sample sizes and less conclusive data.
